# Making Big Data Useful for Health Care: A Summary of the Inaugural MIT Critical Data Conference

**DOI:** 10.2196/medinform.3447

**Published:** 2014-08-22

**Authors:** Omar Badawi, Thomas Brennan, Leo Anthony Celi, Mengling Feng, Marzyeh Ghassemi, Andrea Ippolito, Alistair Johnson, Roger G Mark, Louis Mayaud, George Moody, Christopher Moses, Tristan Naumann, Vipan Nikore, Marco Pimentel, Tom J Pollard, Mauro Santos, David J Stone, Andrew Zimolzak, MIT Critical Data Conference 2014 Organizing Committee

**Affiliations:** ^1^MIT Critical Data Conference 2014 Organizing CommitteeInstitute for Medical Engineering & ScienceMassachusetts Institute of TechnologyCambridge, MAUnited States

**Keywords:** big data, open data, unreliable research, machine learning, knowledge creation

## Abstract

With growing concerns that big data will only augment the problem of unreliable research, the Laboratory of Computational Physiology at the Massachusetts Institute of Technology organized the Critical Data Conference in January 2014. Thought leaders from academia, government, and industry across disciplines—including clinical medicine, computer science, public health, informatics, biomedical research, health technology, statistics, and epidemiology—gathered and discussed the pitfalls and challenges of big data in health care. The key message from the conference is that the value of large amounts of data hinges on the ability of researchers to share data, methodologies, and findings in an open setting. If empirical value is to be from the analysis of retrospective data, groups must continuously work together on similar problems to create more effective peer review. This will lead to improvement in methodology and quality, with each iteration of analysis resulting in more reliability.

##  Introduction

Failure to store, analyze, and utilize the vast amount of data generated during clinical care has restricted both quality of care and advances in the practice of medicine. Other industries, such as finance and energy, have already embraced data analytics for the purpose of learning. While such innovations remain relatively limited in the clinical domain, interest in “big data in clinical care” has dramatically increased. This is due partly to the widespread adoption of electronic medical record (EMR) systems and partly to the growing awareness that better data analytics are required to manage the complex enterprise of the health care system. For the most part, however, the clinical enterprise has not had to address the problems particular to “big data” because it has not yet satisfactorily addressed more fundamental data management issues. It is now becoming apparent that we are on the cusp of a great transformation that will incorporate data and data science integrally within the health care domain. In addition to the necessary major digital enhancements of the retrospective analyses that have variably been in place, real time and predictive analytics will also become ubiquitous core functionalities in the more firmly data-based environment of the (near) future. The initial Massachusetts Institute of Technology (MIT) Critical Data Conference was conceived and conducted to address the many data issues involved in this important transformation [1,2].

Increasing interest in creating the clinical analog of “business intelligence” has made evident the necessity of developing and nurturing a clinical culture that can manage and translate data-based findings, including those from “big data” studies. Combining this improved secondary use of clinical data with a data-driven approach to learning will enable this new culture to close the clinical data feedback loop facilitating better and more personalized care. Authors have noted several hallmarks of “big data”: very large datasets, a large number of unrelated and/or unstructured datasets, or high speed or low latency of data creation [3,4]. The intensive care unit (ICU) provides a potent example of a particularly data rich clinical domain with the potential for both clinical and financial benefits if these large amounts of data can be harnessed and systematically leveraged into guiding practice. Thus, we use the term “Critical Data” to refer to big data in the setting of the ICU.

This paper summarizes the lectures and group discussions that took place during the recent Critical Data Conference at MIT, Cambridge MA, on January 7, 2014. The conference was the second part of a two-part event that brought together clinicians, data scientists, statisticians and epidemiologists.

**Figure 1:**
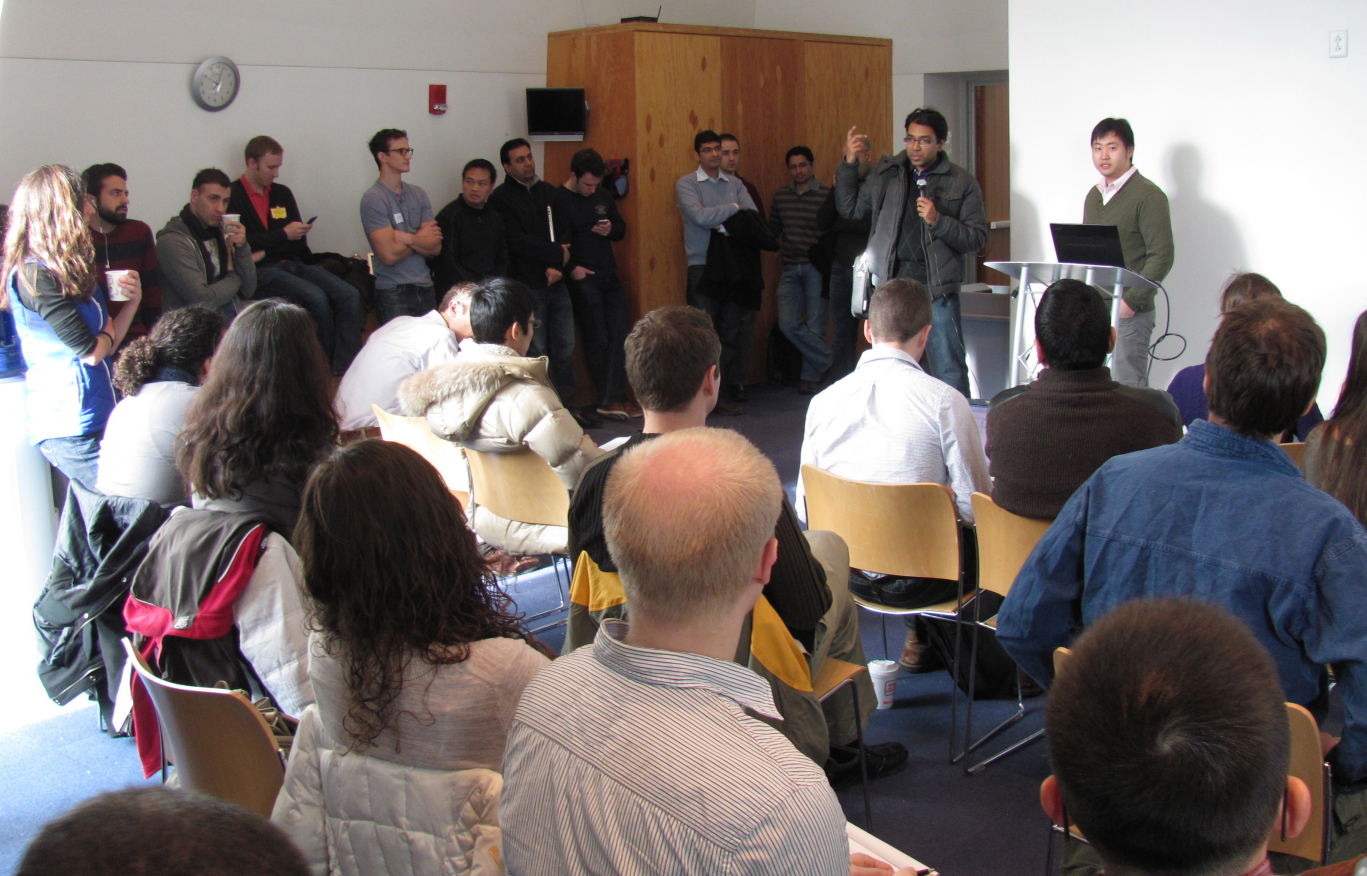
Presentation at the Critical Data Marathon. Photo credit: Andrew Zimolzak.

The event opened with a “data marathon” on January 3-5, 2014 (Figure 1), which brought together teams of data scientists and clinicians to mine the Multiparameter Intelligent Monitoring in Intensive Care (MIMIC) database (version II). MIMIC II is an open-access database consisting of over 60,000 recorded ICU stays from the adult intensive care units at the Beth Israel Deaconess Medical Center (BIDMC) in Boston, MA [5]. Over 100 people participated in the two-day data marathon, and posters of the projects were displayed at the Critical Data Conference.

**Figure 2:**
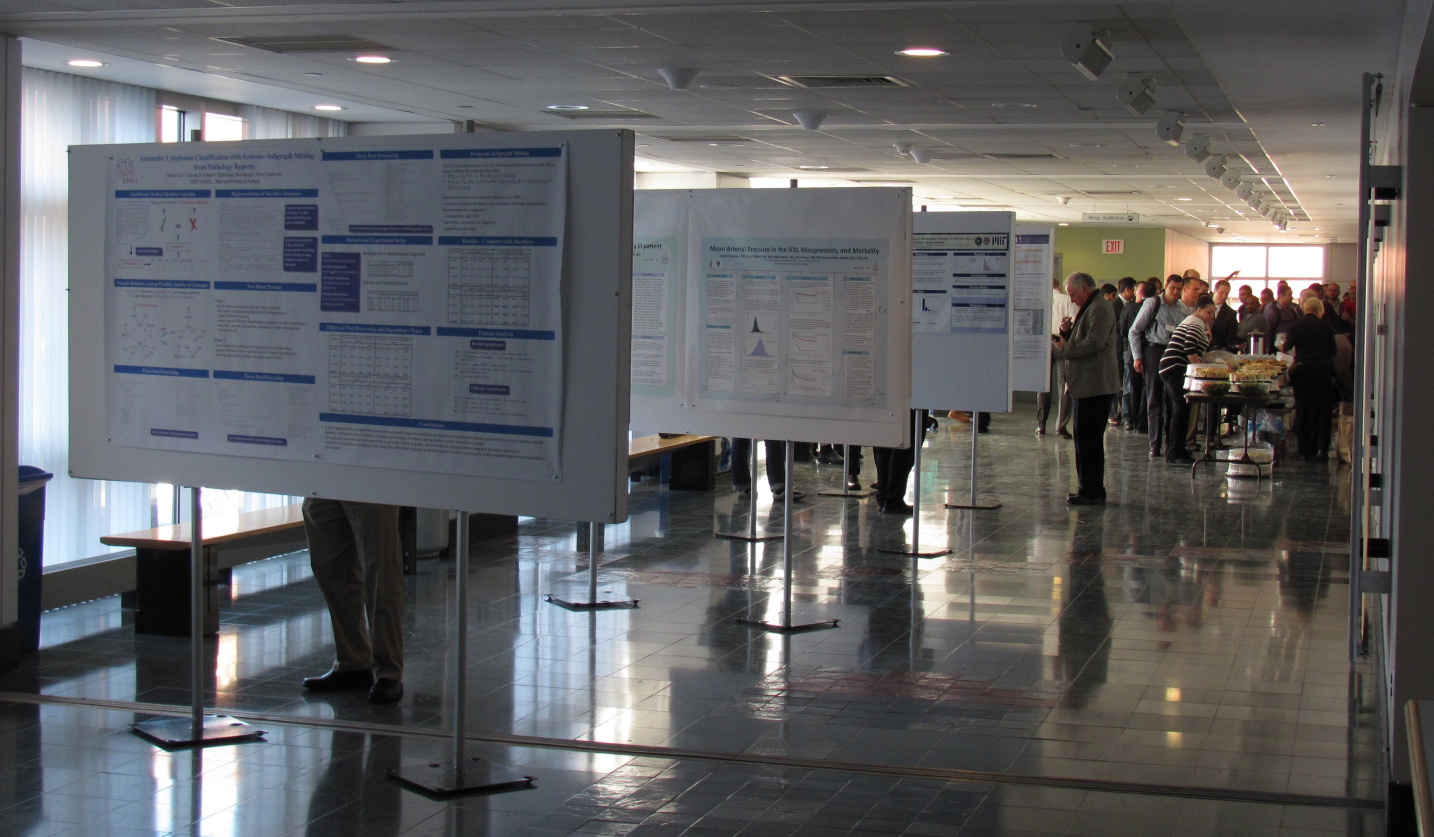
Critical Data poster session. Photo credit: Andrew Zimolzak.

The Critical Data Conference on January 7 was an approximately ten-hour program comprising two keynote addresses (Jeffrey Drazen, MD and John Ioannidis, MD, PhD), seven individual lectures, three panel discussions, and two poster sessions (Figure 2). The overall conference theme was meaningful secondary use of big data from critical care settings. Materials from the conference (program, slides, and videos) are available online at the MIT Critical Data conference site [6].

## The Problem

In his keynote address, Jeffrey Drazen, MD, Editor-in-Chief of the New England Journal of Medicine, noted that the number of evidence-based recommendations built on randomized controlled trials (RCTs), the current gold standard for data quality, is insufficient to address the majority of clinical decisions. Subsequently, clinicians are often left to practice medicine “blindly.” Without the knowledge generation required to capture the decisional factors involved in realistic clinical scenarios, clinical decision making is often less data-driven than determined by the “play of chance” buttressed by past experience. Historically, a doctor took a history, performed a physical examination and made a diagnosis based on what he or she observed. As technology and medical theory progressed, knowledge such as laboratory and imaging modalities helped mitigate chance in the diagnosis of disease. Rote application of existing knowledge is not enough, as physicians want to establish causality. Until now this has been done with theories, but moving forward, theories will be inadequate unless they are confirmed, translated to practice, and systematically disseminated in clinical practice.

This trial-and-error process continues today because data generated from routine care is most often not captured and is rarely disseminated for the purpose of improving population health. Even in information-rich care settings like the ICU, the knowledge necessary to mitigate the play of chance is lacking [7,8]. As such, the ICU provides a fertile ground for potential improvement. Specifically, Drazen suggested a potential role for clinical data mining to answer questions that cannot be answered using RCTs [9]. This approach would likely yield benefits both more quickly and with fewer resources.

Drazen concluded with the question “At what point is data good enough?” Documented associations may be strong but not sufficiently “proven” to establish causality. Drazen drew a comparison with experimental physicists who hone future studies on the work of theorists as well as on prior experimental results: biomedical informaticians can identify meaningful associations that can then guide design of new RCTs where data quality can be increased by controlling for potential confounders. This will require cross-disciplinary collaboration of frontline clinicians, medical staff, database engineers and biomedical informaticians, in addition to strong partnerships with health information system vendors in order to close the loop from knowledge discovery during routine care to the real time application of best care for populations.

## Secondary Usage of Clinical Data

Charles Safran, MD, MS, Chief of the Division of Clinical Computing at the BIDMC and Harvard Medical School, spoke next, sharing the dream of evidence-based medicine (EBM): the ideal situation in which quality evidence would exist to guide clinicians through all the conundrums faced on a near-daily basis (eg, which test to order, how to interpret the test results, and what therapy to institute). For the last half-century, prospective RCTs have been the gold standard in EBM. Safran noted, as Drazen had, that such trials suffer from a number of limitations including economic burdens and design limitations. An RCT can only address a severely limited bundle of particularly well-posed clinical questions. For many clinical situations, it is either unethical or even impossible to proceed with an RCT. Furthermore, the inclusion and exclusion criteria often limit the generalizability of an RCT study and, given the time it normally takes to run an RCT, it is very difficult for these studies to remain current with the rapidly evolving practice of medicine.

Can another approach avoid at least some of the limitations of RCTs? Safran suggested that retrospective observational studies (ROS) utilizing EMR data are a promising avenue for generating EBM. Digital records contain extensive clinical information including medical history, diagnoses, medications, immunization dates, allergies, radiology images, and laboratory and test results. Consequently, routinely collected EMR data contains the rich, continuous and time-sensitive information needed to support clinical decision making and evidence generation [10]. However, despite the many potential benefits, Safran pointed out that secondary use of EMR data is still subject to limitations: EMR data were not collected primarily for the purpose of evidence generation and data analytics but for real-time and longitudinal patient care [11]. As a result, EMR data are often poorly structured, disorganized, unstandardized, and contaminated with errors, artifacts, and missing values.

Safran echoed one of Drazen’s points by proposing that we should combine the usage of prospective RCTs and ROS in such a way that each complements the limitations of the other. Furthermore, he suggested the possibility of incorporating additional novel sources of data such as social media data, health data from portable sensors, and genetic data. While there are many barriers to establishing such a comprehensive framework, a big data picture of clinical, genetic, and treatment variables holds promise in revolutionizing diagnosis and treatment.

## Connecting Patients, Providers, and Payers

For John Halamka, MD, MS, the Chief Information Officer of BIDMC, working with big data in hospital systems is hugely challenging but at the same time holds tremendous promise in providing more meaningful information to help clinicians treat patients across the continuum of care. In his position, Halamka has been tasked to aggregate data in novel ways in order to provide better care for BIDMC’s patient population. One opportunity for furthering “big data in health care” is to normalize the data collected via their EMR system and store it in large, centralized databases. In turn, analytic tools can then be applied to identify and isolate the quality data reporting measures required to participate as an Accountable Care Organization (ACO) under the Affordable Care Act.

Halamka emphasized that building these large datasets does not intrinsically provide value from the start, stating that “workflow is disparate, the vocabulary is disparate, and the people are disparate.” Therefore, the normalization of data and its distillation into standard schemas are difficult due to discrepancies across longitudinal data. Further, since each vendor models concepts differently, there must be an emphasis on developing a “least common denominator” concept map across vendors’ offerings.

Nevertheless, through this normalization effort, doctors can utilize “scorecards” to evaluate their own patient population within and across the different payment models, such as Blue Cross Blue Shield’s Alternative Quality Contact measures, the Center for Medicare and Medicaid (CMS) Physician Quality Reporting System measures, and the CMS ACO measures. In addition, physicians can query this dataset to identify the most effective treatment regimes. However, such queries do pose privacy and security issues in the hospital setting, and these risks are further complicated by hospital staff utilizing personal mobile devices such as cell phones, laptops, and tablets.

## Creating a Data-Driven Learning System

**Figure 3:**
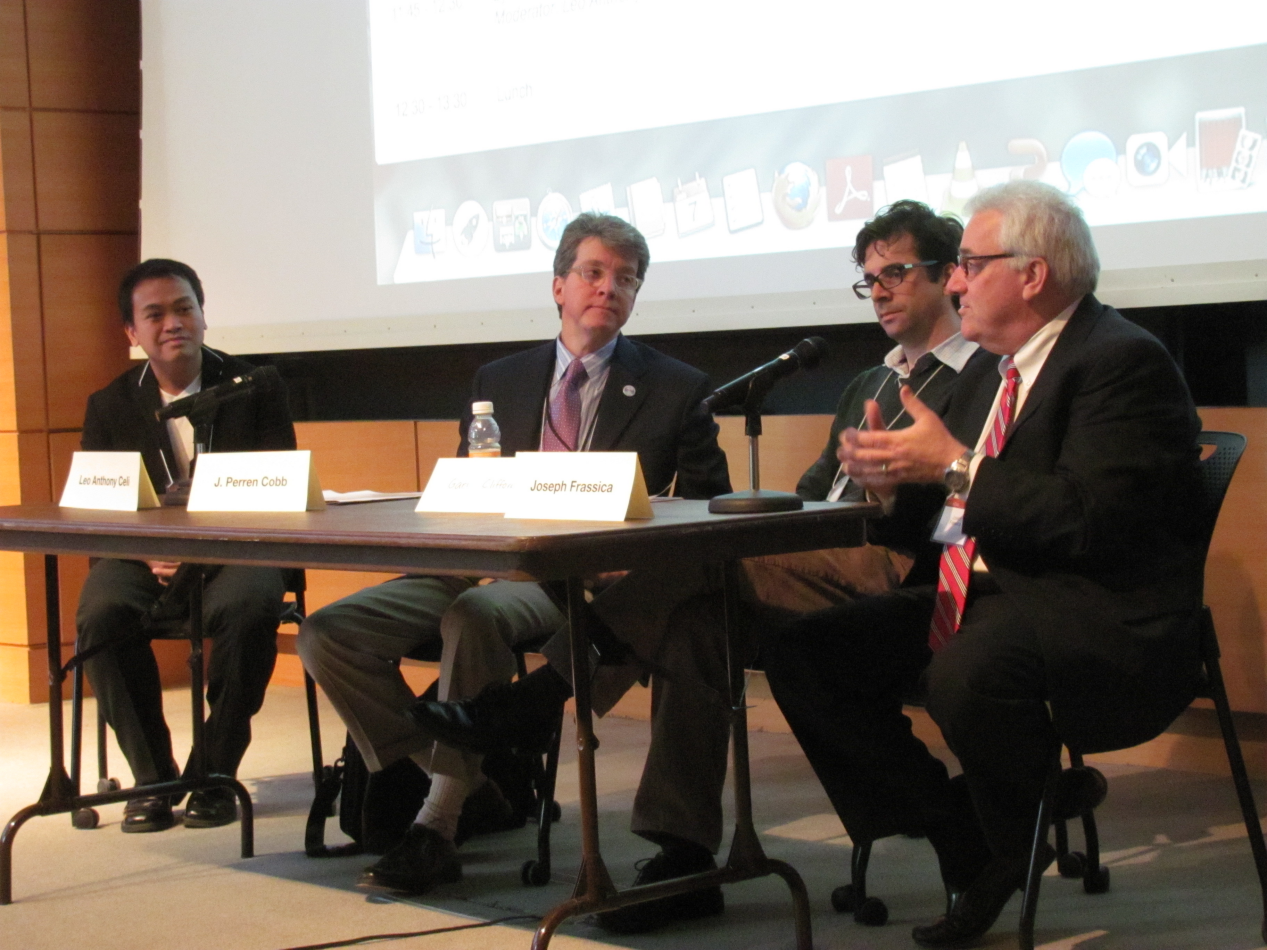
Data-driven learning system panel. Photo credit: Andrew Zimolzak.

The problem posed to the first panel (Figure 3), comprising Gari Clifford, PhD, Perren Cobb, MD, and Joseph Frassica, MD, and moderated by Leo Anthony Celi, MD, MS, MPH, was how to create a data-driven learning system in clinical practice [8]. Privacy concerns were cited as the central barrier, as there is a tradeoff between re-identification risk and the value of sharing. Furthermore, recent work shows patients are reluctant to share for certain purposes such as marketing, pharmaceutical, and quality improvement measures, indicating a need for public education about the benefits of data sharing and that shared data can be utilized without being used for marketing and other unwanted purposes [2].

There is also tension between intellectual property rights and transparency. Resolution of this may require collaboration between government, industry and academic institutions, as seen with the US Critical Illness and Injury Trials Group [12]. There is also a risk that data sharing will make authors reluctant to write audacious or unconventional papers (as did Reinhart and Rogoff [13]), if data sharing puts such papers at perceived higher risk of refutation (such as the refutation of Herndon et al [14]).

Finally, the panel raised concerns about the high quantity but perceived low quality of the data that is actually captured. While there is hope that automatically captured data may be more accurate than manually entered data, there is also some risk that doing so will introduce additional noise, furthering the problem of quantity over quality. This concern poses the challenge of capturing more and higher quality data in order to promote reproducibility. Panelists observed that multidisciplinary conferences like the Critical Data Conference are especially beneficial in this regard, as they provide an opportunity for clinicians and data scientists to better understand the relation between real-world activity and the data that such activity generates.

## Physician Culture as a Barrier to Spread of Innovation

**Figure 4:**
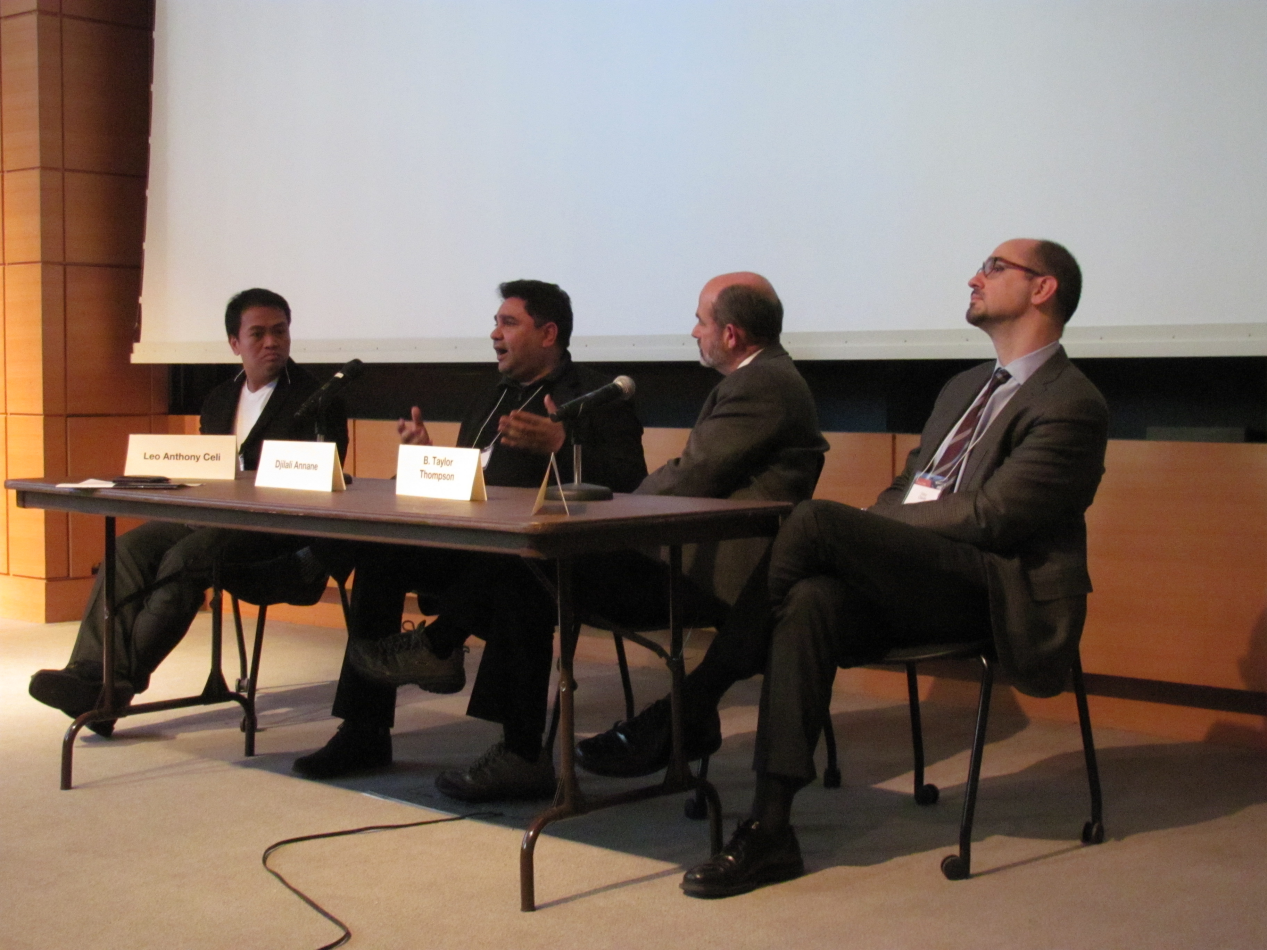
Physician culture panel. Photo credit: Andrew Zimolzak.

In the following panel moderated by critical care physician Leo Anthony Celi, MD, MS, MPH, fellow intensivists Djillali Annane, MD, PhD, Peter Clardy, MD and Taylor Thompson, MD reflected on the barriers presented by the current clinician culture toward the goal of data-driven innovation in medicine (Figure 4). The panelists observed that historically, EBM was perceived to be incompatible with well-established observational trials and experience, perhaps instilling a residual degree of resistance. Consequently, echoing Safran’s sentiments, it will be increasingly important that “big data” is understood as a complement to RCTs and (patho)physiologic studies. Furthermore, condensing and filtering the vast quantity of data to make it applicable at the bedside will be key to adoption. The specific inclusion of clinicians during the design process will help to deter the creation of tools that inundate staff with extraneous information and burdensome extra tasks. Likewise the incorporation of “big data” into medical education, in a way that students and resident trainees will be able to understand its importance in both everyday care and expediting research, is vital.

While the panel agreed that more evidence is required to determine whether big data can facilitate comparative effectiveness research, it was acknowledged that it is necessary to investigate this alternative since RCTs do not, and will not, provide answers to an important fraction of the decisions required on a daily basis. Scaling up RCTs to account for the thousands of decisions each day is not feasible, so big data approaches may provide the most effective way to fill these gaps. For example, three groups currently leading clinical trials research in the analysis of fluid resuscitation in critically ill patients have collaborated to create a common database architecture to allow for individual patient meta-analysis and for these trials to be evaluated in aggregate by an external monitoring committee.

## The Role of Industry in the Data Revolution in Health Care

There is concern that industry continues to view data as a potential source of revenue and would therefore be opposed to providing open access to what they consider to be proprietary data with business value. In the last panel discussion of the day, moderated by Ambar Bhattacharyya, MBA of Bessem Venture Partners, industry panelists Josh Gray, MBA of AthenaResearch, Enakshi Singh, MS of SAP, and Omar Badawi, PharmD, MPH of Philips Healthcare provided their insights on the topic.

They first addressed the issue of sharing databases freely, noting that concerns associated with data ownership are not restricted to industry. Similar problems and conflicts are observed among most stakeholders in health care data ownership: patients, hospitals, providers, payers, vendors, and academia. Generally speaking, industrial data owners want to protect their data from those who may use it competitively against them, share or sell the data to derive direct clinical value, or profit from possible insights. They also wish to avoid the overhead costs associated with sharing. They are interested in allowing society to leverage their data in order to make gains if, and only if, these other interests remain unaffected.

The costs for responsibly sharing secondary clinical data are not trivial. Although understanding the complexity of the data presents a significant challenge, understanding the workflow for entering data in the primary system is often even more complicated, requiring extensive support. Therefore, sharing secondary clinical data can be a costly initiative for industry, lowering its priority as a business objective. These challenges are further exacerbated when collaboration requires intellectual property agreements. Lack of an accepted standard practice for research agreements, coupled with an outdated patent system, creates barriers to collaboration that are rarely overcome. Many ideas for collaboration either take years to initiate or never come to fruition, due to challenges with developing de novo legal research agreements.

While industry and researchers are not philosophically opposed to sharing data to ensure reproducibility, protections from the aforementioned concerns are critical. Can the data be shared without the risk of lost intellectual property? If not, the incentives for innovation may be minimized. Who will bear the costs for ensuring that the replicating team fully understands the nuances of the data? Who will prevent competitors or others with malicious intent from inappropriately labeling valid research as “junk science”? Such underhanded interventions could introduce confusion around valid earlier findings and unfairly distract and denigrate the primary researchers.

Ultimately, there is a growing sense that data will become less of a commodity over time if governments continue to support the development and maintenance of open access research networks. As the scale and quality of these surpass those of privately owned databases, society will benefit as the obstacles to collaboration and the value of retaining private ownership diminish.

## The Unreasonable Effectiveness of Data

Peter Szolovits, PhD from the MIT Computer Science and Artificial Intelligence Lab, highlighted how big data can often trump good, but smaller, data. Researchers at Google have been making a similar argument from a decade-long experience with natural language processing, showing that for some important tasks an order of magnitude growth in the size of a dataset leads to improvements in performance that can overshadow improvements in modeling technique [15]. They have also argued that discarding rare events is a bad idea because although these may be individually rare, they could prove to be significant later when examined on a much larger scale.

In the clinical world, patient state depends on complex pathophysiology dictated by genetic predispositions, environmental exposures, treatments, and numerous other factors. While, there are many potential ways to formulate clinical outcomes into complex statistical models, it is often the simple models that give the best, and most interpretable, results. Some clinicians and epidemiologists have already used large sources of observational data to improve clinical practice, especially in identifying drug side-effects, for example, for rosiglitazone [16], and rofecoxib [17]. Cox proportional hazard, naïve Bayes, linear and logistic regression, and similar models can use aggregated variables to summarize dynamic variation without adding additional complexity.

## The Story of MIMIC: Open-Access Critical Care Data

Since researchers who seek to create new clinical knowledge and tools are dependent upon the availability of relevant data, restricting access to data introduces barriers that stifle research progress. This simple principle has been at the heart of the research of Roger Mark, MD, PhD since the 1980s, a time when his work was focused on developing real-time arrhythmia analysis tools for use in patient monitoring.

Like today, the norm for researchers in the 1980s was to privately maintain closed databases for their own benefit. So when Mark’s team needed data, they began the painstaking work of creating their own resource, collecting electrocardiograms from patients at Boston’s BIDMC and in the process, adding over 100,000 annotations. Breaking from tradition, they openly shared the dataset, reasoning that the more people who analyzed it, the better the overall understanding of arrhythmias would become. This dataset become known as the MIT-Beth Israel Hospital (MIT-BIH) Arrhythmia Database [18].

The consequences were far-reaching. Not only did the MIT-BIH Database stimulate research interest, it generated beneficial competition and became a shared resource for evaluating algorithms. Researchers competed to see whose work performed best on the standard data, eventually leading to the database becoming part of a federal requirement for evaluation of commercial algorithms. This success led the team to develop further resources unique in their openness, including PhysioNet, a platform for open physiologic data, and MIMIC II, a rich database of critical care data.

PhysioNet has over 50,000 registered users in over 120 countries and international recognition for accelerating the pace of discovery [19]. Mark attributes much of PhysioNet’s success to the progressive mindset of the participating collaborators. Success has required not only funding, but also a collaborative approach among partnering clinicians, researchers, hospital technologists, and local ethics committees. Participation of commercial partners was also required, and obtained, in order to decrypt the proprietary data formats output by their monitoring systems.

Reproducibility of research and open data are increasingly getting the attention they deserve, but changing practice requires support at all levels [8]. For open technology to be embraced, funders must recognize the added value from a robust database infrastructure and allocate funds accordingly. Researchers too must embrace open approaches that perhaps challenge some of the underlying career reward systems. With changing attitudes, and by engaging the creative energy of the worldwide research community, Mark’s hope is that MIMIC will become a multinational resource leading to the generation of new knowledge and new tools.

## Opportunities and Challenges in Wearable Sensor Datasets

The ability to create and capture data is exploding and offers huge potential for health organizations around the world to save both lives and scarce resources. Yadid Ayzenberg, PhD discussed the “Opportunities and Challenges in Wearable Sensor Data” in his talk, focusing on how the combination of wearable technology and the near ubiquitous access to mobile phones have the potential to address some of the challenges in health care. Examples include the works of Poh et al [20] and Sano and Picard [21], which used a wrist-worn electrodermal activity and accelerometry biosensor for detection of convulsive seizures and sleep stages.

Wearable technologies provide a way to transition from a traditional aperiodic “snapshot” monitoring approach to a continuous and longitudinal monitoring paradigm, increase patients’ engagement in their care, and facilitate doctor-patient interactions. Already massive amounts of personal health data are being generated through consumer devices such as mobile phones and wristbands that monitor sleeping patterns, exercise, stress, calorie consumption and more. In most instances, however, the data are stored on a per-device basis, and there are unsolved issues concerning data management, ownership, privacy, and misuse. The noise and artifacts in the data measured by wearable sensors also present an important challenge. New analytic methods that transform “dirty data” into good quality data are needed.

## Big Data, Genomics, and Public Health

According to Winston Hide, PhD, the promise of a new economy based on data-driven discovery and decision-making is also motivating his own field of genomics. Advances in genome sequencing technology will allow the cost of sequencing a genome to be less than $100 in the near future. Consequently, it is estimated that by 2015, one million genomes will be digitally available with high expectations for public health benefits. However, Hide cautions that there is still a “genome variants” problem to be solved. This was exemplified by the case of Kira Peikoff who was predicted to have a 20% above average risk of developing psoriasis by one commercial sequencing product, while predicted to have a 2% below average risk for the same condition by another [22].

Variations in the reporting of genomic characteristics have two potential root causes. First, there is a sampling problem caused by the use of different sequencing technologies, which introduces errors in evaluating genome assembly. Second, there is an interpretation problem in defining the role of genes in disease which compromises the prediction of clinical outcomes as determined by the single-nucleotide polymorphism analysis tools.

The availability of millions of genomes will allow completion of the catalogue of genes associated with a particular disease in genome-wide association studies. However, Hide maintains that finding clear drug targets requires the creation of an evolving catalogue of functions which would interpret complex gene pathways [23], and the selection of cohorts that would not only depend on ethnicity (the classic phenotype), but also on physiological and even molecular differences.

The application of genomic tests to public health will contribute to the transformation of physicians into data-centric specialists and pave the way for “precision medicine” [24]. This challenging way of delivering health care calls for new strategies and tactics for translating research into clinical practice. These will likely include the creation of open-access genomic and clinical databases, use of a common scientific language [25] and (open) data access tools. Regulatory bodies, such as the Food and Drug Administration, will have a role in guaranteeing the standardization and reliability of diagnostics based on genetic tests. These tools must guarantee the reproducibility [8] of discovered genome signals and contribute to the improvement of online platforms that map genetic features to diseases and their treatment (eg, Cancer Genome Atlas; PharmGKB).

## The Pitfalls and Potential of Big Data in Health Care

Proponents of big data have made grandiose claims of expanding human knowledge by orders of magnitude through empirical analysis and data mining, but as Stanford professor John Ioannidis, MD, PhD says, “with big data comes big problems”. Ioannidis discussed the darker side of data analysis, in which bias has led a large proportion of published medical science to come to the wrong conclusion. Author of the most downloaded paper in PLOS Medicine, “Why Most Published Research Findings Are False” [26], Ioannidis argued that most statistically significant results are likely to be false positives. For example, using the national drug and cancer registry database of Sweden, Ioannidis and colleagues found that almost one third of the 560 medications evaluated in isolation were associated with a higher cancer risk.

As Ioannidis highlights, the issue is not in the quantity of data we have. Increasing sample sizes is a huge boon to the medical field. The issue resides in a lack of transparency. When he reviews a paper published in a journal on a new dataset, his thoughts immediately drift to those studies that were not published. This is quantified in the so-called “vibration of effects”, where depending on the confounding variables for which adjustments are made, completely opposite conclusions can be drawn. For vitamin E, for example, adjusting for a certain subset of confounders led to the conclusion that it increases the relative risk of mortality, whereas adjusting for another equally plausible set of confounders gave the opposite result, that is, a reduction in mortality risk. This may explain why 90% of effects in RCTs were lower in subsequent published trials [27].

## Comparative Effectiveness Using Big Data

Limitations aside for now, ROS do provide an opportunity to conduct comparative effectiveness studies on research questions that would be unlikely to be examined by an RCT, or would be inherently biased if an RCT were conducted. The illustrative example presented by Una-May O’Reilly, PhD was the question of the potential benefit of diuretic use to accelerate removal of fluids given during resuscitation in ICU patients who have recovered from sepsis. Retrospective analysis would be easily marred by “selection bias”. In fact, if patients are allocated (not randomized) to two groups, treatment and non-treatment, it is very likely that the allocation would be done on the basis of patient condition and biased by clinical severity resulting in unreliable results.

In a ROS, the data consists of a series of days during which the treatment was administered (D+) or not (D-). Because these decisions were being made on a daily basis, it is even harder to capture the covariance structure. O’Reilly refers to this as the “Non-Decision Day Dilemma”. In order to deal with this, the covariance structure has to account for time-varying information with respect to a specific day. It is easy to take the treatment day as a reference and align all patients who received treatments with respect to this event (D+). For non-treated patients, aligning time-series is more complicated as every day is essentially a “non-decision day”. Considering every single day would result in a widely unbalanced dataset where the length-of-stay influences the individual contribution of each patient. To account for this, it is possible to randomly sample N negative days (D*-) and pair them up with the positive instances (D+) based on a statistical similarity criterion with respect to the time from admission. This is achieved by defining a propensity score for each patient for every day during their ICU stay. The propensity score thus enables appropriate cohort matching such that comparative effectiveness can be appropriately assessed.

This modest example illustrates the sort of robust and reliable statistical technique that evidence-based medicine requires. It can reduce sample noise and improve the reliability of conclusions, and it leads toward methodology standardization across studies. Beyond these local improvements, meta-studies will also be a requirement to validate any local finding. These are only possible with data sharing and open data initiatives such as the MIMIC-II initiative. One strength of this database is the dual culture and scientific activity it generates because data science can only fully benefit from collaboration between data scientists and domain experts (in this case, intensivist physicians).

## Conclusions

Although the future of “big data” in health care remains unclear, its role will be undeniably important. This conference was effective in collating the broad range of perspectives on the many challenges facing EBM in the 21st Century. As several speakers suggested, one possible opportunity is to adopt a pragmatic approach to EBM, combining RCT and ROS. This combination may employ ROSs to fill the gaps where it is impractical, unlikely or impossible to conduct a RCT or to drive hypothesis generation for further RCT analysis.

It is also crucial to acknowledge that any ROS requires a multidisciplinary approach, integrating clinical knowledge with a broad range of data analytic skills ranging from biostatistics, machine learning, and signal processing to data mining. Encouraging a change in physician culture can likely be accomplished through updating education programs as well as by creating centers for excellence that can showcase the impact of ROS to the broader medical fraternity. These centers for excellence should host open, transparent, easily accessible data warehouses, which will facilitate study reproducibility and allow for a new wave of collaborative learning. Only by understanding the potential biases of any analysis, and fostering a system of normative data sharing, will the medical community be able to gain reliable knowledge from data, and produce research findings that do not turn out to be false.

## Abbreviations

ACO: accountable care organization
BIDMC: Beth Israel Deaconess Medical Center
CMS: Center for Medicare and Medicaid
EBM: evidence-based medicine
EMR: electronic medical record
ICU: intensive care unit
MIMIC: Multiparameter Intelligent Monitoring in Intensive Care
MIT: Massachusetts Institute of Technology
MIT-BIH: Massachusetts Institute of Technology – Beth Israel Hospital
RCT: randomized controlled trial
ROS: retrospective observational study
