# Metadata Correction: Making Big Data Useful for Health Care: A Summary of the Inaugural MIT Critical Data Conference

**DOI:** 10.2196/medinform.4226

**Published:** 2015-01-21

**Authors:** Omar Badawi, Thomas Brennan, Leo Anthony Celi, Mengling Feng, Marzyeh Ghassemi, Andrea Ippolito, Alistair Johnson, Roger G Mark, Louis Mayaud, George Moody, Christopher Moses, Tristan Naumann, Vipan Nikore, Marco Pimentel, Tom J Pollard, Mauro Santos, David J Stone, Andrew Zimolzak, MIT Critical Data Conference 2014 Organizing Committee

**Affiliations:** ^1^MIT Critical Data Conference 2014 Organizing CommitteeInstitute for Medical Engineering & ScienceMassachusetts Institute of TechnologyCambridge, MAUnited States

The author Vipan Nikore, MD, MBA was inadvertently omitted from the list of authors during the submission process of the paper "Making Big Data Useful for Health Care: A Summary of the Inaugural MIT Critical Data Conference" (JMIR Med Inform 2014;2(2):e22). The author Vipan Nikore, MD MBA (MIT Critical Data Conference 2014 Organizing Committee, Institute for Medical Engineering & Science, Massachusetts Institute of Technology, Cambridge, MA, USA) should have been added after Tristan Naumann, MS in the original published manuscript. The missing author has been added to the original online paper with publishing this correction notice on January 21, 2015, and the correct full-text has been resubmitted to Pubmed Central and other full-text repositories.

